# Dramatic radiotherapy response in a necrotic lymphoma mass: a case report

**DOI:** 10.1186/s13256-020-02438-1

**Published:** 2020-07-28

**Authors:** Nicholas McNeil, Peter Gorayski, Danielle Blunt, Daniel Roos

**Affiliations:** 1 Royal Adelaide Hospital, Adelaide, SA Australia; 2 University of South Australia, Adelaide, SA Australia; 3 School of Medicine, University of Adelaide, Adelaide, SA Australia

**Keywords:** Lymphoma, Palliative, Radiotherapy, Radiosensitivity

## Abstract

**Background:**

Diffuse large B-cell lymphoma (DLBCL) represents the most common form of non-Hodgkin lymphoma and is characterized by an aggressive natural history. It often presents with rapid symptom development and disease progression. Most lymphomas are inherently radiosensitive, which allows for effective disease control from relatively low radiation doses. We report a case of a dramatic radiotherapy response in a necrotic diffuse large B-cell lymphoma mass in an elderly patient with early-stage diffuse large B-cell lymphoma, illustrating the potential for palliative radiotherapy to reduce disease burden in patients not fit for systemic therapy. There is no current consensus recommendation for radiotherapy treatment in this setting.

**Case presentation:**

A 97-year-old Caucasian woman presented to the emergency department of our institution with a painful, malodorous, necrotic right upper neck mass, which had progressed over a two-month period. Investigations confirmed stage 1A diffuse large B-cell lymphoma. Palliative radiotherapy was delivered to a dose of 25 Gray (Gy) in five fractions on alternate days over two consecutive weeks. After four months, the mass completely resolved with no residual symptoms.

**Conclusion:**

Dramatic responses resulting in durable local control and improvement in quality of life are achievable with palliative radiotherapy, owing to the radiosensitivity of diffuse large B-cell lymphoma.

## Background

Non-Hodgkin lymphoma (NHL) includes over 50 histologic subtypes, of which diffuse large B-cell lymphoma (DLBCL) is the most common form of aggressive lymphoma, representing 30% of cases [1]. It can present with rapid symptom development and disease progression.

DLBCL presents most commonly (65% of cases) with painless, rapidly enlarging lymphadenopathy in the cervical and abdominal regions [2]. Thirty percent of patients also present with B-symptoms, including night sweats, weight loss and fever [2]. The disease can either transform from a low-grade lymphoma or develop as *de novo* high-grade disease [2]. It has a slight male preponderance and median age of 64 years at diagnosis [2].

Gene expression profiling has allowed subclassification of DLBCL by its cell of origin into either the germinal center B-cell (GCB) or activated B-cell (ABC) type. This subtyping has prognostic significance [3], with GCB-DLBCL having better clinical outcomes than ABC-DLBCL [4].

Radiotherapy has been used for many years to effectively treat NHL of both low- and high- grade, in the primary, consolidative, salvage and palliative settings [5]. For localized disease, systemic treatment is combined with radiotherapy [6], which in this setting results in improved progression-free survival and reduced disease recurrence at the primary site [6]. For advanced DLBCL, radiotherapy is used in the management of large volume disease to improve local disease control [6].

Patients are frequently elderly, however, limiting the ability to effectively and safely deliver systemic therapy. Low-dose involved-field radiation therapy of varying dose schedules has been shown to effectively palliate symptoms from advanced lymphoma [6]. Doses as low as 4 Gray (Gy) delivered over two fractions have been shown to result in 50–80% response rates for DLBCL at 21 days [6, 7], with a median time to disease progression following radiotherapy of 12 months [6]. Treatment with hypofractionated schedules (dose > 2 Gy per fraction), including 8 Gy in a single fraction, have also been used in this setting to provide effective symptom control, with minimal toxicity and inconvenience to patients [8, 9]. Higher-dose hypofractionated schedules with total doses of 8–30 Gy are recommended in the International Lymphoma Radiation Oncology Group (ILROG) guidelines [10]. We report a case of a dramatic radiotherapy response in a necrotic lymphoma mass in an elderly patient with early-stage DLBCL, illustrating the potential for single modality palliative radiotherapy to reduce disease burden in patients not fit for systemic therapy.

## Case presentation

**Fig. 1:**
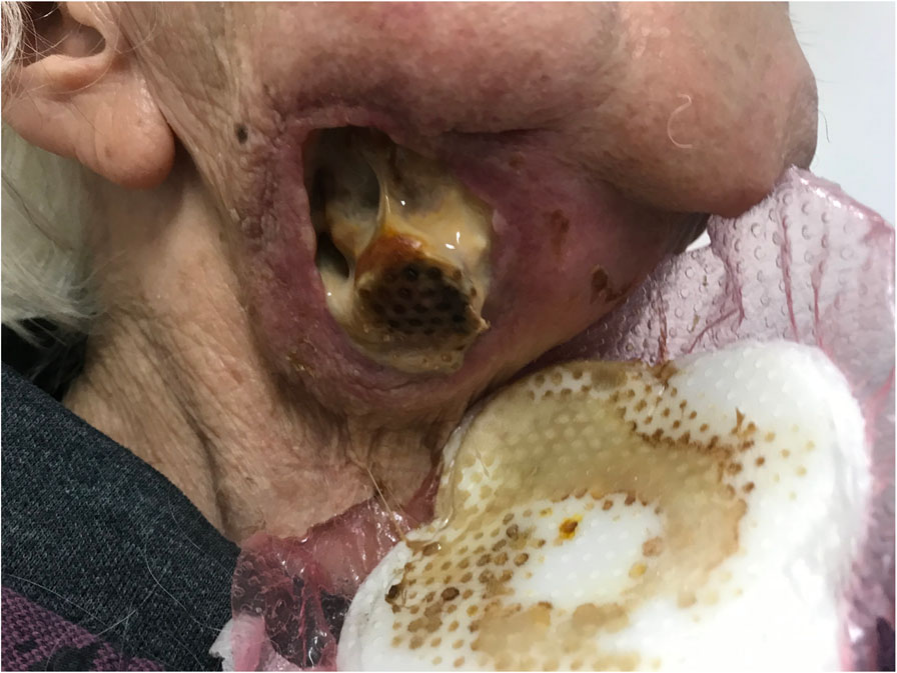
The patient’s right neck at presentation

A 97-year-old Caucasian woman presented to the emergency department of our institution with a painful, malodorous, necrotic right submandibular mass (Fig. 1). A solitary enlarged lymph node had been identified in this region on computed tomography two months prior. She gave no history of B-symptoms, immunodeficiency or immunosuppression.

She lived at home alone with support from a carer who visited twice daily. She did not smoke tobacco or consume alcohol. Her comorbidities included dementia, congestive cardiac failure, paroxysmal atrial fibrillation, a previous above-knee deep vein thrombosis and early-stage breast cancer diagnosed 17 years earlier, managed surgically and considered to be in remission. Her regular medications included bisoprolol 2.5 mg daily, ramipril 1.25 mg daily, aspirin 100 mg daily, digoxin 62.5 μg daily and periciazine 2.5 mg daily.

She was afebrile and hemodynamically normal at presentation and other than the right neck mass, her physical examination was unremarkable. Blood work showed a white cell count of 10.87 × 10^9^/L (normal range, 4–11) with slight neutrophilia at 9.05 × 10^9^/L (normal range, 1.8–7.5) and lymphopenia at 1.01 × 10^9^/L (normal range, 1.5–3.5). Her hemoglobin was normal at 137 g/L, as was her platelet count of 197 × 10^9^/L. Her potassium was measured as 3.3 mmol/L (normal range, 3.5–4.9) but all other electrolytes were within the normal range. Her creatinine measured 69 μmol/L with an estimated glomerular filtration rate of 64 ml/minute/1.73 m^2^. Her lactate dehydrogenase was elevated at 267 U/L (normal range, 110–230) but her liver enzymes were all within the normal range.

**Fig. 2:**
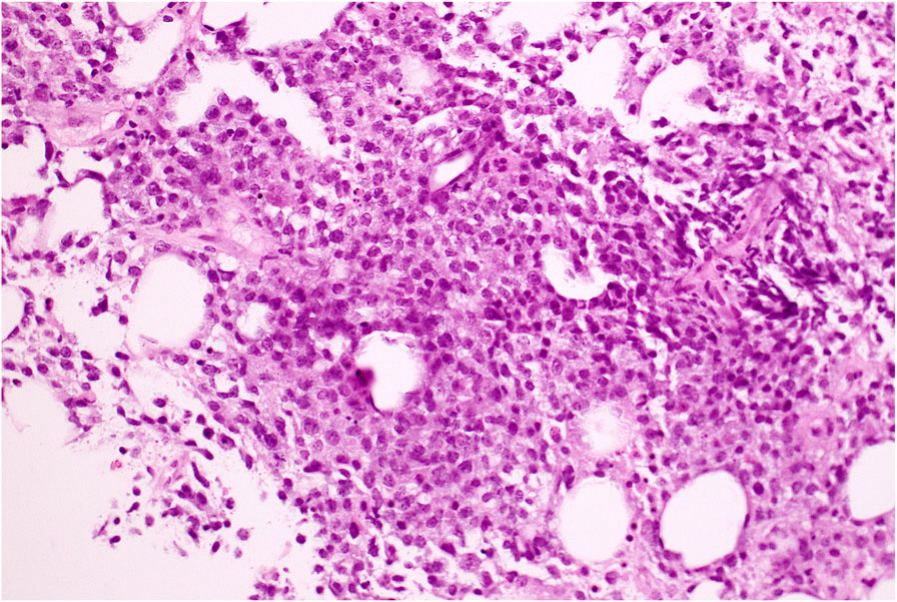
Lymph node core biopsy with hematoxylin and eosin stain showing discohesive large cells with marked cellular pleomorphism and atypia

**Fig. 3:**
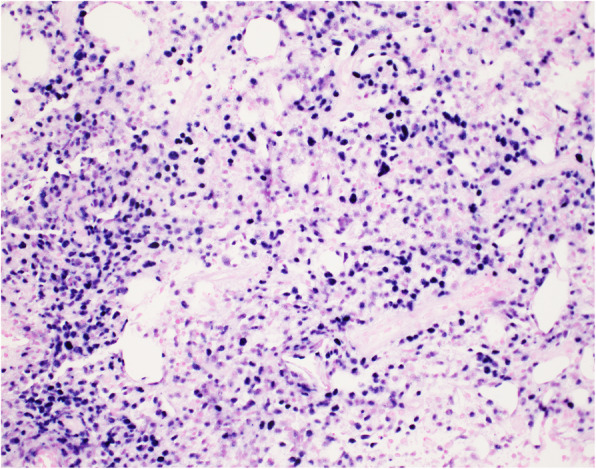
Lymph node core biopsy Epstein Barr encoded RNA *in situ* hybridization showing a positive result

Following non-diagnostic fine needle aspiration of the mass, core biopsy revealed DLBCL with a non-germinal center immunophenotype using the Han's algorithm, and Epstein Barr encoded ribonucleic acid (RNA) *in situ* hybridization positivity (Figs. 2 and 3).

**Fig. 4:**
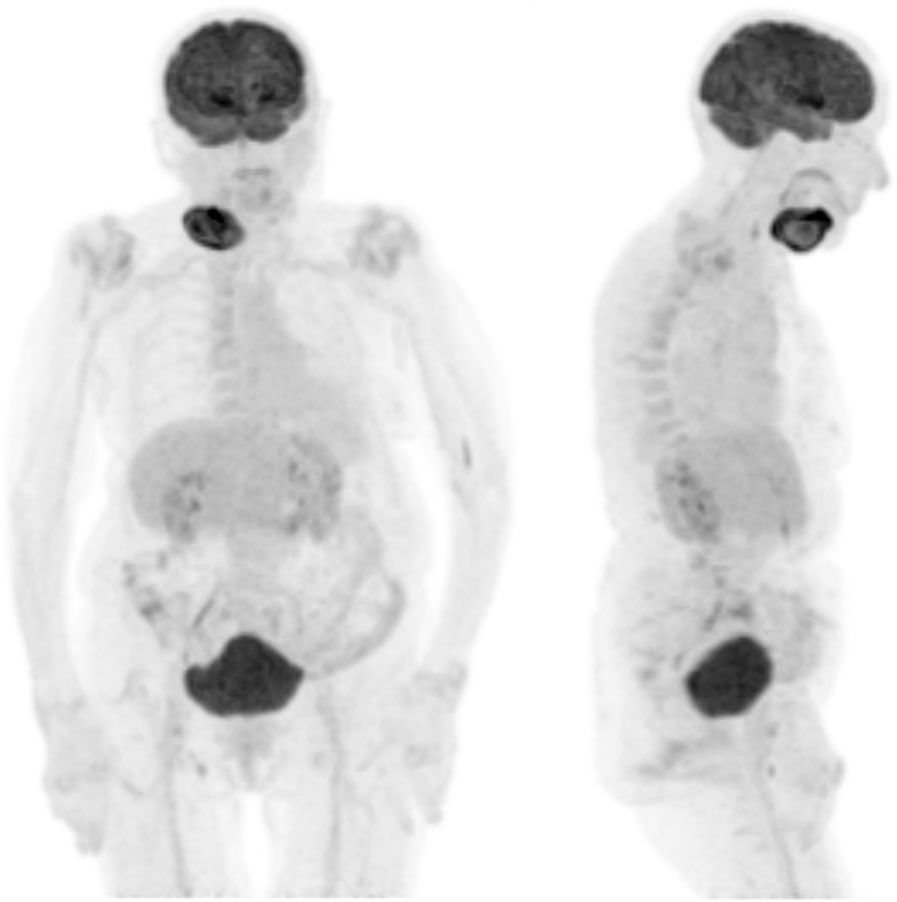
18-FDG positron emission tomography maximum intensity projection images with anterior (left) and lateral (right) views showing the avid right submandibular mass to be the only site of disease

Computed tomography and 18-fluorodeoxyglucose (FDG) positron emission tomography (Fig. 4) revealed this to be the only site of disease, consistent with stage 1A.

Given her age, frailty, performance status and multiple comorbidities, she was deemed unfit for systemic therapy and palliative radiotherapy was recommended.

She received a dose of 25 Gy delivered over five fractions on alternate days over a two-week period. Treatment was delivered using a four-beam three-dimensional conformal technique with 6-megavoltage photons and daily image guidance. The treatment incorporated the use of bolus material placed over the surface of the mass to optimize the radiation dose delivered superficially to the skin.

**Fig. 5:**
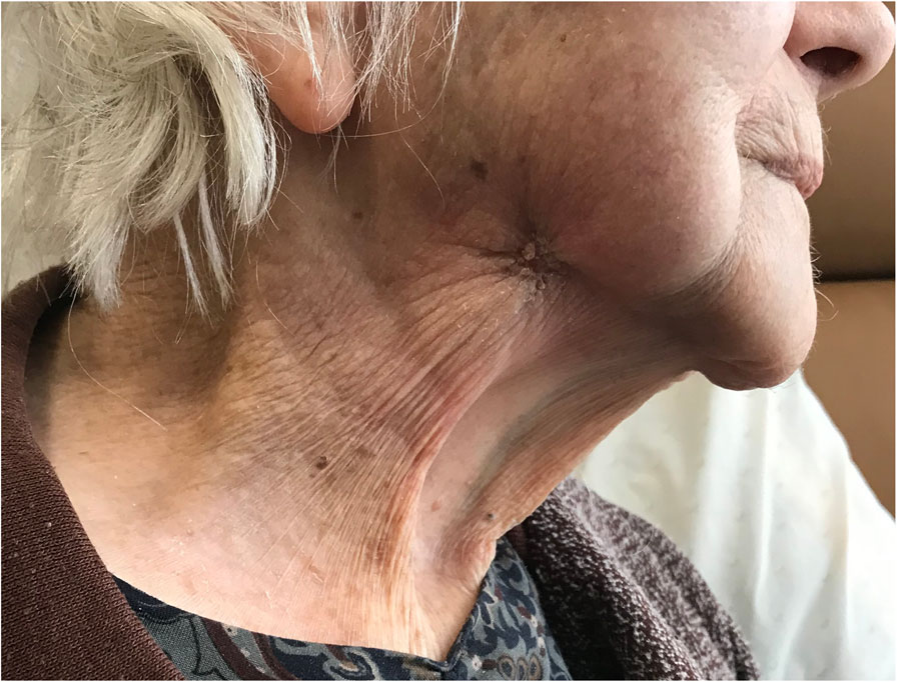
The patient’s right neck four months after completion of radiotherapy

The patient was followed up monthly and after four months the mass completely resolved (Fig. 5). The overlying skin on the right neck had healed to cover the previous necrotic deficit and no mass was palpable. She was asymptomatic with no observable toxicity at the latest review at four months.

## Discussion

Multimodal therapy incorporating combinations of chemotherapy, rituximab, and radiotherapy now form the basis of curative and palliative management of early-stage and advanced DLBCL [5].

In this case of an elderly woman of poor performance status, a hypofractionated course of palliative radiotherapy alone was used to treat her early-stage DLBCL. This was in part done to minimize the logistical issues relating to appointment attendance and also to allow delivery of a biological dose higher than traditional low-dose palliative treatment, with the intention of providing more robust local disease control. The dose of 25 Gy delivered over five fractions that was used represents a calculated radiobiologically equivalent dose in 2-Gy fractions (EQD2) of 31.25 Gy, for the accepted tumor α/β ratio of 10 (α/β ratio represents the relative sensitivity of a tissue type to radiation).

In early-stage DLBCL (stage I–II), radiation therapy has an important role in increasing local disease control as part of combined therapy, to consolidate the effect of systemic therapy [11]. The National Comprehensive Cancer Network (NCCN) guidelines recommend total doses of 30–36 Gy following a complete response to systemic therapy and 40–50 Gy following a partial response [12].

For patients with DLBCL who are unsuitable for systemic therapy, single-modality radiotherapy used as primary treatment has been shown to provide effective local control [11]. Specifically in the setting of relapsed or refractory disease in patients unfit for systemic treatment, the ILROG guidelines recommend radiotherapy with curative intent to locoregionally confined disease to a total dose of 45–55 Gy, delivered in doses of 1.8–2 Gy per fraction [10].

In the palliative setting, the role of radiotherapy is well established for the multimodal treatment of refractory or relapsed DLBCL, or as single-modality treatment for those unfit for systemic therapy. Low-dose radiotherapy, accepted as doses ≤8 Gy delivered in one or more fractions, has been shown in a number of trials to be effective in providing local disease control [6–8]. Brady *et al.*, in their 2016 trial, compared the palliative radiotherapy schedules of 8 Gy delivered in a single fraction with 4 Gy in one or two fractions and showed no significant difference in overall response rates [8]. For this purpose, the ILROG guidelines support the use of hypofractionated schedules of 8–30 Gy, depending on the adjacent dose-limiting normal structures [10].

Wong *et al*., in their 2018 population-based retrospective review of the efficacy of palliative radiation therapy for DLBCL, showed it to be effective for both managing symptoms and providing local disease control [13]. They reviewed 217 patients with a mean age 76 years who received a total of 370 courses of palliative radiotherapy for DLBCL. The median dose delivered had an EQD2 of 19 Gy, considerably lower than the EQD2 of 31.25 Gy in the treatment of our patient. Symptom resolution was reported in 42% of patients overall and following each individual treatment course, 51% of patients reported pain resolution and 36% reported pain improvement. Local control six months after treatment was achieved in 65% of patients. The authors did not identify a significant association between the EQD2 dose of the delivered treatment and the rate of local control achieved.

There is no consensus recommendation for a specific dose-fractionation schedule in the setting of single modality primary radiotherapy for early-stage DLBCL. In this case, patient, tumour and treatment factors, tumor and treatment factors were all considered in determining the high palliative dose of 25 Gy in five fractions, which proved highly effective in providing local disease control.

## Conclusion

We report a remarkable response to radiotherapy in an elderly patient with early-stage DLBCL, illustrating the potential for palliative radiotherapy to reduce disease burden in patients otherwise not fit for systemic therapy. Importantly, lymphomas are among the most radiosensitive cancers, with the radiotherapy dose tailored to the performance status of the individual, which results in minimal toxicity in the majority of cases.

## Data Availability

Not applicable.

## Abbreviations

ABC: Activated B cell
DLBCL: Diffuse large B-cell lymphoma
EQD2: Equivalent dose in 2 Gy per fraction
FDG: Fluorodeoxyglucose
GCB: Germinal center B-cell
Gy: Gray
ILROG: International Lymphoma Radiation Oncology Group
NCCN: The National Comprehensive Cancer Network
NHL: Non-Hodgkin lymphoma
RNA: Ribonucleic acid
